# Supplementary material for: 2D Abrupt Nano‐Junctions Blending sp–sp 2 Bonds on Atomically Precise Heterostructures
Source: Adv Mater. 2026 Aug 23;38(59):e74740. doi: 10.1002/adma.74740 (PMC13689904; doi:10.1002/adma.74740)
Supplement: Supplementary file 1 [file ADMA-38-e74740-s002.pdf]

SUPPORTING INFORMATION

2D Abrupt Nano-Junctions Blending *sp*–*sp*^{2} Bonds on Atomically Precise Heterostructures

Alice Cartoceti*[,1,9] | Simona Achilli*[,2,3,9] | Masoumeh Alihosseini[2] | Adriana E. Candia[4] | Enrico Beltrami[2] | Paolo D'Agosta[1] | Alessio Orbelli Biroli[5] | Francesco Sedona[6] | Andrea Li Bassi[1] | Jorge Lobo-Checa*[,7,8] | Carlo S. Casari*[,1]

[1]Department of Energy, Politecnico di Milano, via R. Lambruschini 6, Milano, 20156, Italy | [2]Department of Physics 'Aldo Pontremoli', Università degli Studi di Milano, via G. Celoria 16, Milano, 20133, Italy | [3]INFN Sezione di Milano and 'European Theoretical Spectroscopy Facility' (ETSF), via G. Celoria 16, Milano, 20133, Italy | [4]Instituto de Física del Litoral, Consejo Nacional de Investigaciones Científicas y Técnicas, Universidad Nacional del Litoral (IFIS-Litoral, CONICET-UNL), Santa Fe, 3000, Argentina [5]Department of Chemistry, Università di Pavia, via T. Taramelli 12, Pavia, 27100, Italy | [6]Department of Chemical Sciences, Università degli Studi di Padova, via F. Marzolo 1, Padova, 35131, Italy | [7]Instituto de Nanociencia y Materiales de Aragon (INMA), CSIC-Universidad de Zaragoza, Zaragoza, 50009, Spain | [8]Departamento de Física de la Materia Condensada, Universidad de Zaragoza, Zaragoza, 50009, Spain | [9]These authors contributed equally to this work. |

Correspondence: Alice Cartoceti (alice.cartoceti@polimi.it) | Simona Achilli (simona.achilli@unimi.it) | Jorge Lobo-Checa (jorge.lobo@csic.es) | Carlo S. Casari (carlo.casari@polimi.it)

Keywords: 2D *sp*–*sp*^{2} carbon heterostructures | On-surface synthesis | Graphdiyne-Graphene interfaces | Carbon nanoelectronics | Density functional theory | Transmission function

S1 | On-Surface Synthesis of hGDY-aGNR Heterostructure

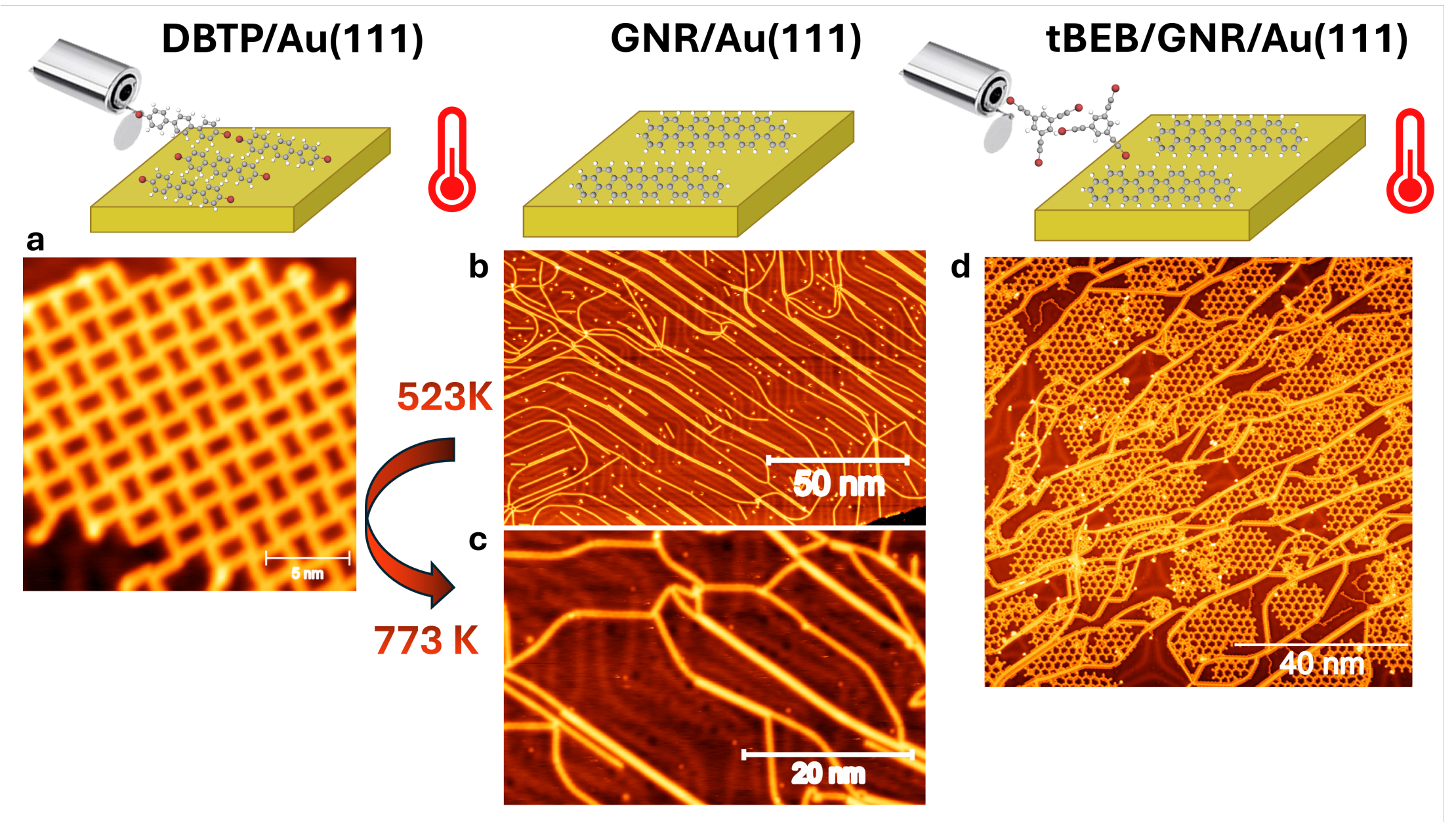

FIGURE S1 | Schematic representation of the on-surface synthesis of lateral hGDY-aGNR heterostructures. For each step, a LT-STM image of the sample is reported. (a) As-deposited DBTP molecules on Au(111) at room temperature. (b) PPP chains formed upon annealing of the sample at 523 K. (c) aGNRs obtained upon annealing of the sample at 773 K. (d) Lateral heterostructures obtained upon the evaporation of tBEB molecules on aGNRs and sample annealing at 400 K. STM setpoint: (a) −10 mV / 100 pA, (b) 100 mV / 50 pA, (c) 300 mV / 50 pA, (d) −50 mV / 10 pA.

S2 | Polydispersity of aGNRs Width

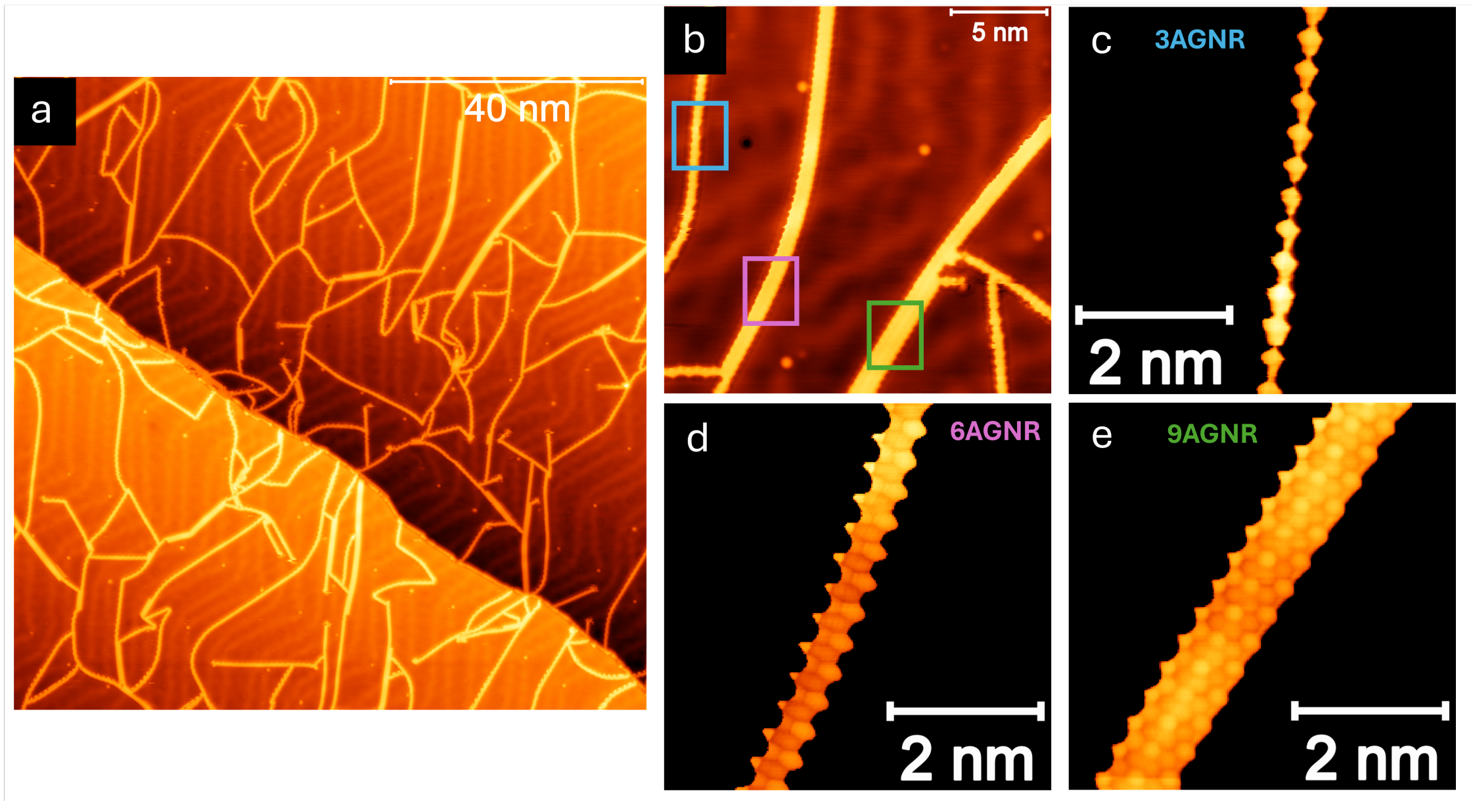

FIGURE S2 | Large scale (a) and atomically resolved (b-e) LT-STM image of aGNRs on Au(111). (c-e) Close-up of the squared regions in (b), namely corresponding to 3-aGNR (blue square, c), 6-aGNR (pink square, d) and 9-aGNR (green square, e). STM setpoint: (a) −20 mV / 100 pA, (b) −10 mV / 130 pA, (c) −2 mV / 60 pA, (d) −2 mV / 60 pA, (e) −2 mV / 60 pA.

S3 | hGDY Termination and Bond Dissociation Energy

We consider two possible terminations of the freestanding hGDY in a rectangular unit cell, namely one with only one Au atom and another one with a terminal Au-Br complex. The systems are periodic along the horizontal direction. We evaluated the stability of the two models by considering the total energy difference between a hGDY fragment terminated with Au-Br and only with Au, by including in the energy balance the contribution of the Br atoms removed. The calculated quantity also furnishes the dissociation energy of the Au-Br complex at the termination of the hGDY. We found that the Au-Br terminated case is 3.40 eV more stable than the Au-terminated hGDY, thus we can conclude that the Au dangling bonds of the hGDY have the tendency to saturate with Br atoms.
To calculate the dissociation energy of C-Au we compared the total energy of the infinite metalated hGDY with that of the Au-terminated, and we identified this difference as the energy required to break C-Au bonds, once the number of atoms in this computation has been properly considered.

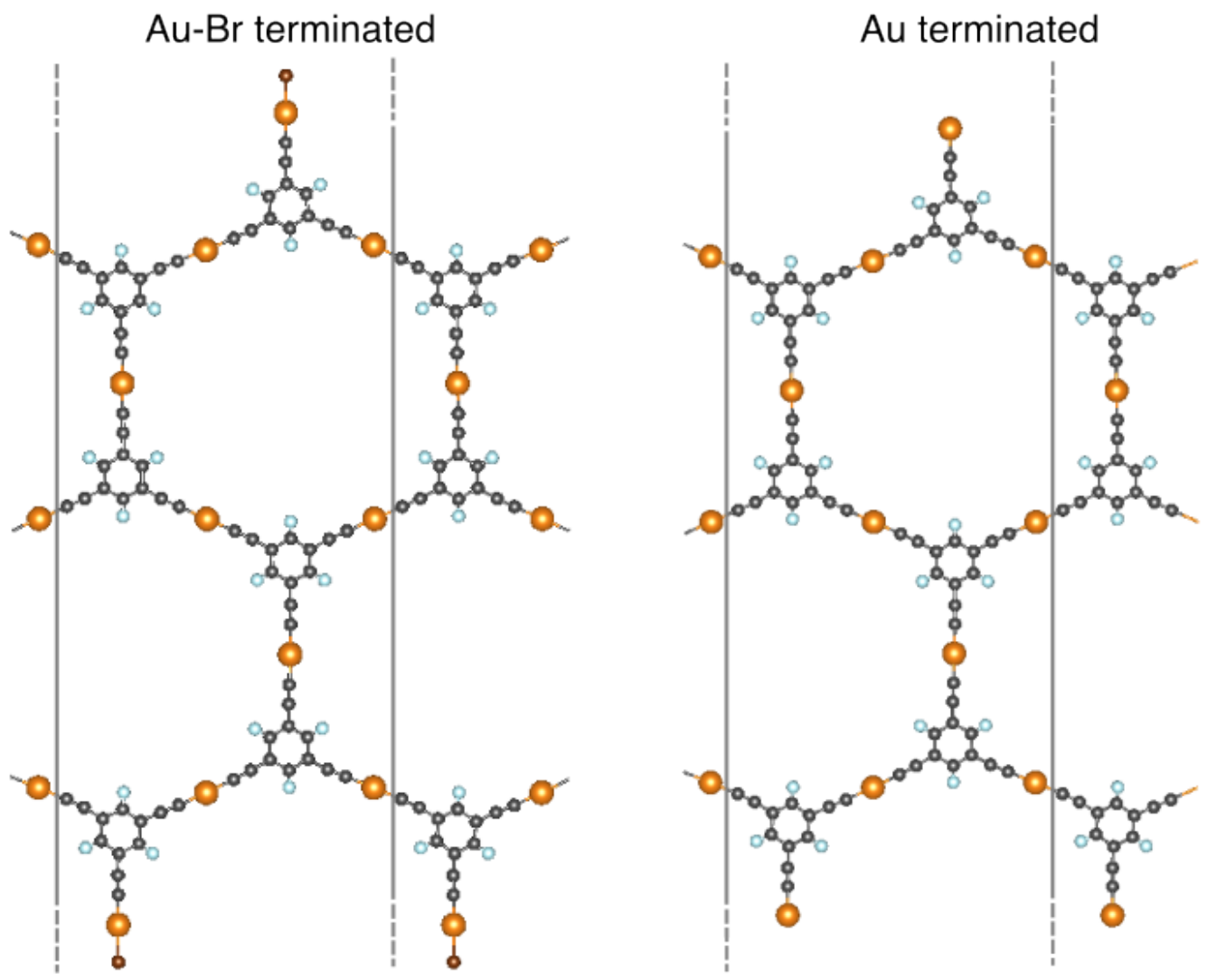

FIGURE S3 | Models of hGDY terminated with Br-Au complex (left) or only Au (right). The horizontal periodicity is marked by the two vertical gray lines corresponding to segments of the unit cell edges. In the vertical direction, the two structures face a vacuum region of tens of angstroms to guarantee the decoupling between the replicas.

S4 | A and B Linking Configurations Between hGDY and aGNRs

The statistical prevalence of the A configuration over the B configuration can be explained by the most favorable matching of the two sublattices in the former. Indeed, in A configuration, the periodicity of the hGDY differs from the one of the aGNR of ∼2.2%, while in the B configuration this difference is of ∼5%. As a consequence the B configuration is 2.58 eV less stable than configuration A.
In both configurations, upon linking, the two subsystems rearrange leading to a compression of the aGNR along the armchair direction and an expansion of the hGDY in the horizontal direction.

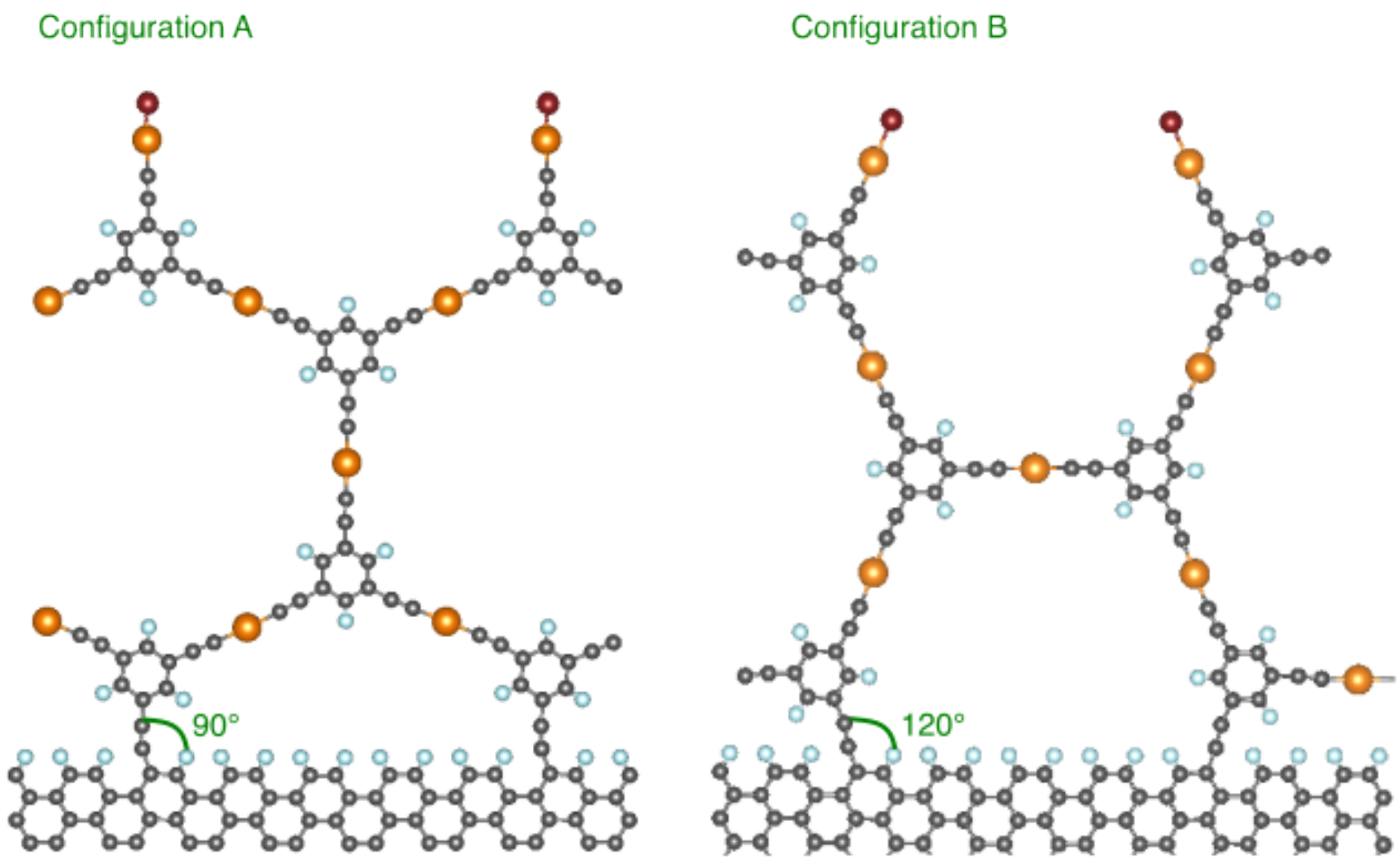

FIGURE S4 | Ball-and-stick atomic model of the two possible configurations, called for simplicity "A" and "B".

S5 | Statistical Method for Determining the A- and B-type Linkage Efficiency

Three types of samples are considered to monitor the linking efficiency between the hGDYs and the aGNRs as a function of the surface density of Br atoms:

1. Br surface density of $0.53 \pm 0.05\ nm^{-2}$ for the standard tBEB coverage.
2. Br surface density of $0.19 \pm 0.07\ nm^{-2}$ upon atomic hydrogen dosage.
3. Br surface density of $0.84 \pm 0.09\ nm^{-2}$ for increased tBEB coverage.

Br atoms' surface density is obtained by considering their averaged number per unit area, using LT-STM images acquired in different regions of each of the three samples. Specifically, for each of them:
Sample 1 (standard tBEB coverage): 3 LT-STM images, of which 2 having an area of $30 \times 30\ nm^2$ and 1 having an area of $30 \times 20\ nm^2$. The number of Br atoms per each image is: 447 ($30 \times 30\ nm^2$), 304 ($30 \times 20\ nm^2$), and 523 ($30 \times 30\ nm^2$).
Sample 2 (after hydrogen dosage): 5 LT-STM images, of which 2 having an area of $30 \times 30\ nm^2$, 2 having an area of $35 \times 35\ nm^2$, and 1 having an area of $25 \times 25\ nm^2$. The number of Br atoms per each image is: 106 ($30 \times 30\ nm^2$), 182 ($35 \times 35\ nm^2$), 200 ($35 \times 35\ nm^2$), 247 ($30 \times 30\ nm^2$), and 152 ($25 \times 25\ nm^2$).
Sample 3 (increased tBEB coverage): 6 LT-STM images, of which 3 having an area of $30 \times 30\ nm^2$, 2 having an area of $20 \times 20\ nm^2$, and 1 having an area of $25 \times 25\ nm^2$. The number of Br atoms per each image is: 761 ($30 \times 30\ nm^2$), 723 ($30 \times 30\ nm^2$), 613 ($30 \times 30\ nm^2$), 362 ($20 \times 20\ nm^2$), 385 ($20 \times 20\ nm^2$), and 537 ($25 \times 25\ nm^2$).
Having defined the Br surface density for each of the samples, the efficiency of the A-type and B-type linkages has been obtained considering 169 formed junctions over different regions of the three types of samples listed above. In particular, 8 different regions of three different samples have been considered:
Sample 1: 3 regions, having an area of $30 \times 30\ nm^2$, $30 \times 30\ nm^2$, and $30 \times 20\ nm^2$.
Sample 2: 3 regions, having an area of $30 \times 30\ nm^2$, $30 \times 30\ nm^2$, and $35 \times 35\ nm^2$.
Sample 3: 2 regions, having an area of $30 \times 30\ nm^2$ and $30 \times 30\ nm^2$.
In the following, for each of the analyzed images, the total number of formed junctions is reported, discriminating between those of A-type and those of B-type. The error bar is obtained as the standard error of the binomial distribution:

$$\sigma = \sqrt{\frac{p(1-p)}{N_{\mathrm{tot}}}}, \qquad \text{where} \qquad p = \frac{N_{\mathrm{link}}}{N_{\mathrm{tot}}}. \tag{S1}$$

Sample 1 (standard tBEB coverage)

	Total linking sites	N. A linkages	N. B linkages	Efficiency A	Efficiency B
Image #1	23	19	4	82.61%	17.39%
Image #2	9	7	2	77.78%	22.22%
Image #3	22	16	6	72.73%	27.27%
	**TOT: 54**	**TOT: 42**	**TOT: 12**	77.78%	22.22%

Overall, 54 junctions have been considered, of which 42 are of type A and 12 of type B, resulting in an efficiency of **77.78** $\pm$ **5.6**% for type A linkage and **22.22** $\pm$ **5.6**% for type B linkage. The mean linking efficiency obtained from the three individual LT-STM images is **77.7** $\pm$ **4.9**% for type A linkage, and it is **22.3** $\pm$ **4.9**% for type B linkage, reflecting the reproducibility of the observation across different images. An example of the procedure is reported here below for the sample of type 1, i.e., obtained with the standard tBEB coverage. The green circles represent the A-type linkages, while the black circles represent the B-type linkages.

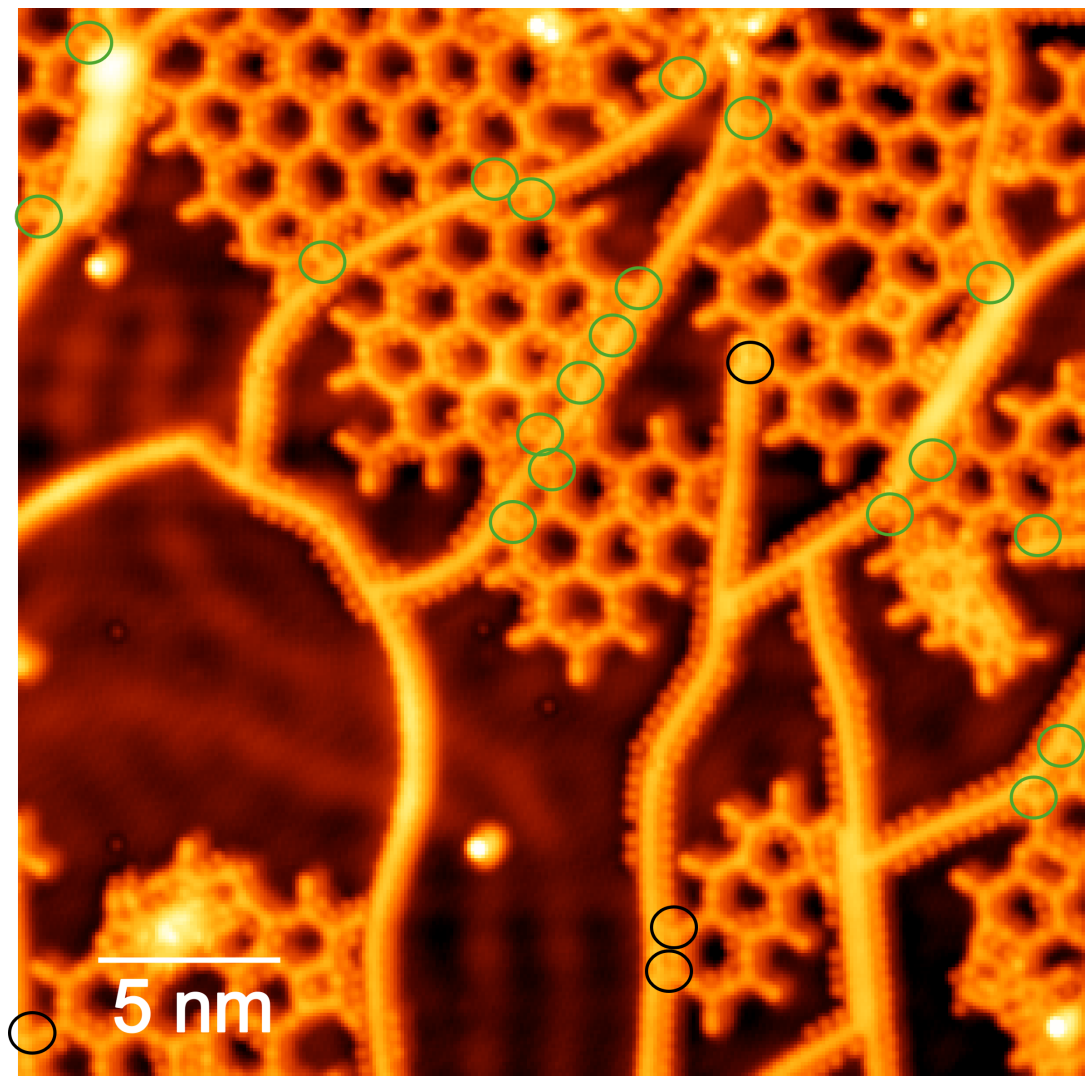

FIGURE S5 | High resolution LT-STM image of a region of the hGDY–aGNR heterostructure on Au(111) upon annealing at 530 K. Green circles represent A-type links. Black circles represent B-type links. STM setpoint: −100 mV / 10 pA.

Sample 2 (after hydrogen dosage)

	Total linking sites	N. A linkages	N. B linkages	Efficiency A	Efficiency B
Image #1	27	21	6	77.78%	22.22%
Image #2	40	35	5	87.50%	12.50%
Image #3	20	16	4	80.00%	20.00%
	**TOT: 87**	**TOT: 72**	**TOT: 15**	82.76%	17.24%

The efficiency is **82.76** $\pm$ **4**% for type A linkage and **17.24** $\pm$ **4**% for type B linkage. The mean linking efficiency obtained from the three individual LT-STM images is **81.76** $\pm$ **5.0**% for type A linkage, and it is **18.24** $\pm$ **5.0**% for type B linkage, reflecting the reproducibility of the observation across different images.

Sample 3 (increased tBEB coverage)

	Total linking sites	N. A linkages	N. B linkages	Efficiency A	Efficiency B
Image #1	19	15	4	78.95%	21.05%
Image #2	9	7	2	77.78%	22.22%
	**TOT: 28**	**TOT: 22**	**TOT: 6**	78.57%	21.43%

The efficiency is **78.57** $\pm$ **7.7**% for type A linkage and **21.43** $\pm$ **7.7**% for type B linkage. The mean linking efficiency obtained from the two individual LT-STM images is **78.36** $\pm$ **0.8**% for type A linkage, and it is **21.64** $\pm$ **0.8**% for type B linkage, reflecting the reproducibility of the observation across different images.

Overall, the number of considered junctions is 169, of which 136 are junctions of type A, while 33 are of type B. Therefore, the percentage of type-A links is **80.47** $\pm$ **3.05**%, while that of type-B links is **19.53** $\pm$ **3.05**%.

S6 | Additional STM Images of the Covalent Bonding

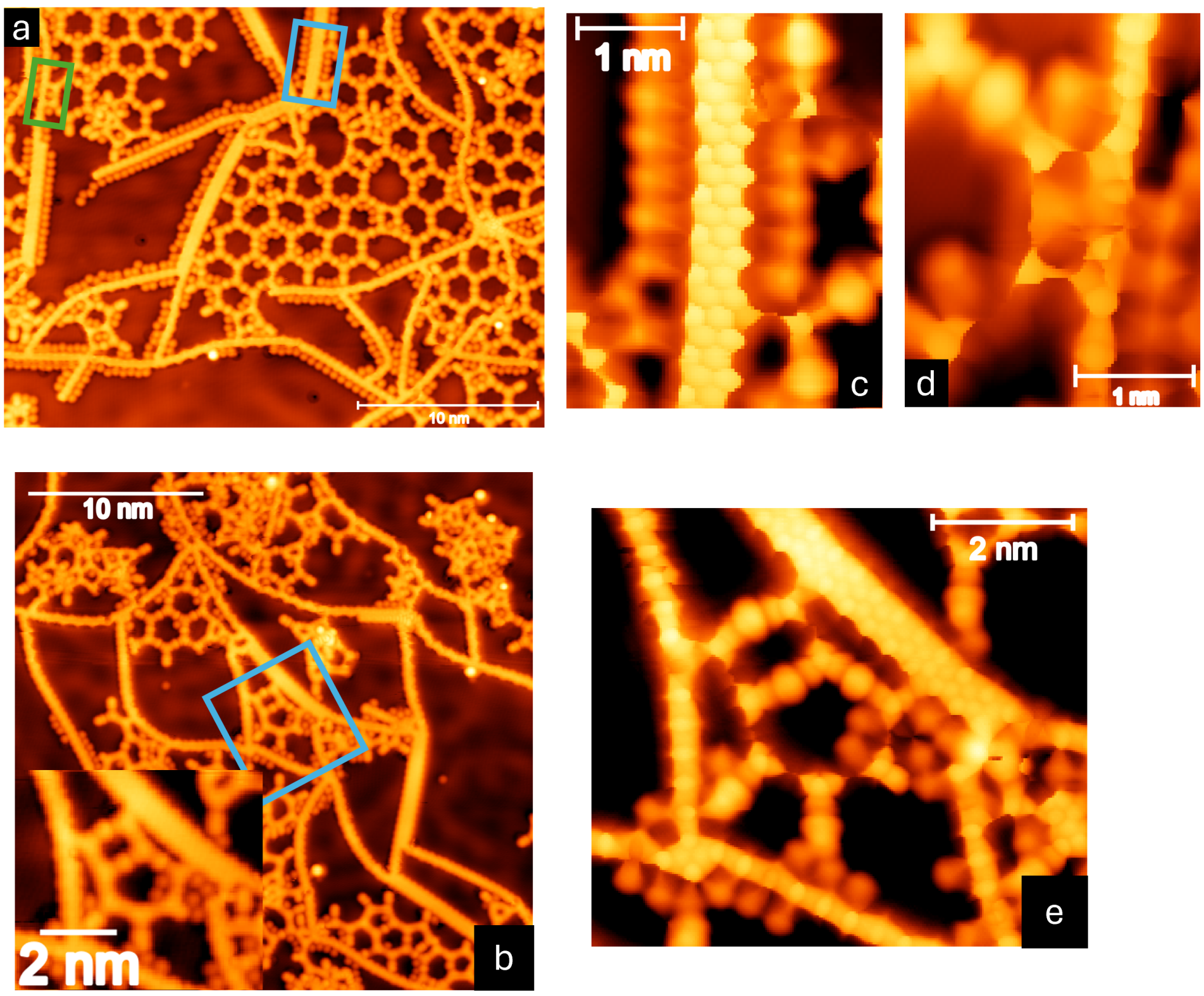

FIGURE S6 | (a,b) Large-scale LT-STM image of the system upon the formation of covalent bonds between hGDY metalated network and 3- and 6-aGNRs. (b,inset) close up of the blue rectangle in (b). Br-functionalized STM tip. (c) Close-up of the blue rectangle in (a). (d) Close-up, flipped by 180°, of the green rectangle in (a). (e) Close up of the blue rectangle in (b). CO-functionalized STM tip. STM setpoint: (a) −10 mV / 80 pA, (b, b inset) −30 mV / 80 pA, (c) −10 mV / 80 pA, (d) −3 mV / 80 pA, (e) −2.7 mV / 50 pA.

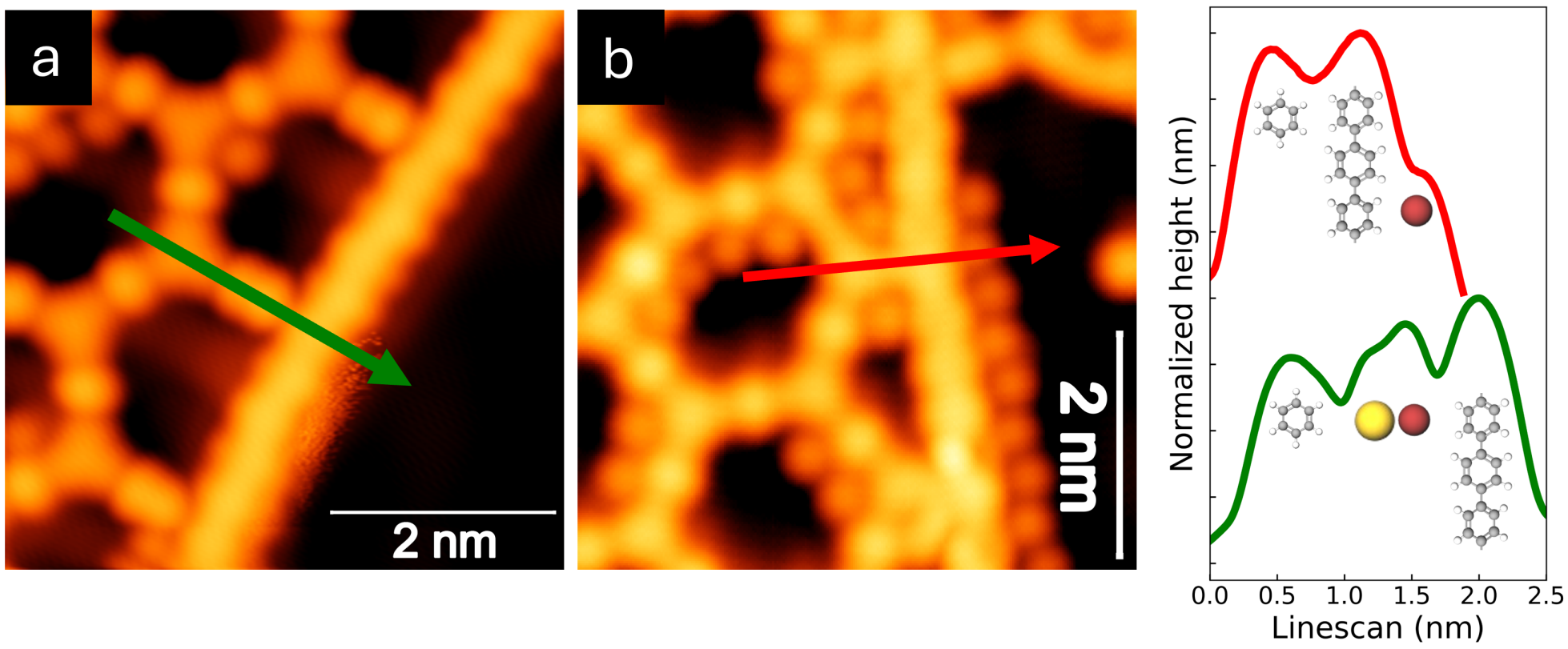

FIGURE S7 | High resolution LT-STM image of the aGNR-hGDY heterostructure on Au(111) before (a) and after (b) the formation of the hetero-junctions, i.e., upon annealing at 400 K and 530 K, respectively. Line profile taken along the green and red arrows on (a) and (b) are shown on the right, together with the ball-and-stick atomic model of benzene ring, gold adatom, Br atom and PPP. STM setpoint: (a) 100 mV / 100 pA, (b) −1 mV / 80 pA.

S7 | Statistical Method for Determining the Linking Efficiency

The procedure employed for determining the surface density of Br atoms is reported in Section S5 for each of the three analyzed samples. In particular, Sample 1 corresponds to the standard tBEB coverage, Sample 2 to the sample obtained after hydrogen dosage, and Sample 3 to that with increased tBEB coverage.
Having defined the Br surface density for each of the samples, the efficiency of the hGDY-aGNR linking process corresponds to the number of junctions effectively formed over the number of "possible" junctions, i.e., those where the hGDY termination is directly facing the aGNR edge, being at a distance equal or slightly larger than a Br atom decorating the nanoribbon. An example of the procedure to extract the linking efficiency is reported here below for the sample of type 1, i.e., obtained with the standard tBEB coverage.

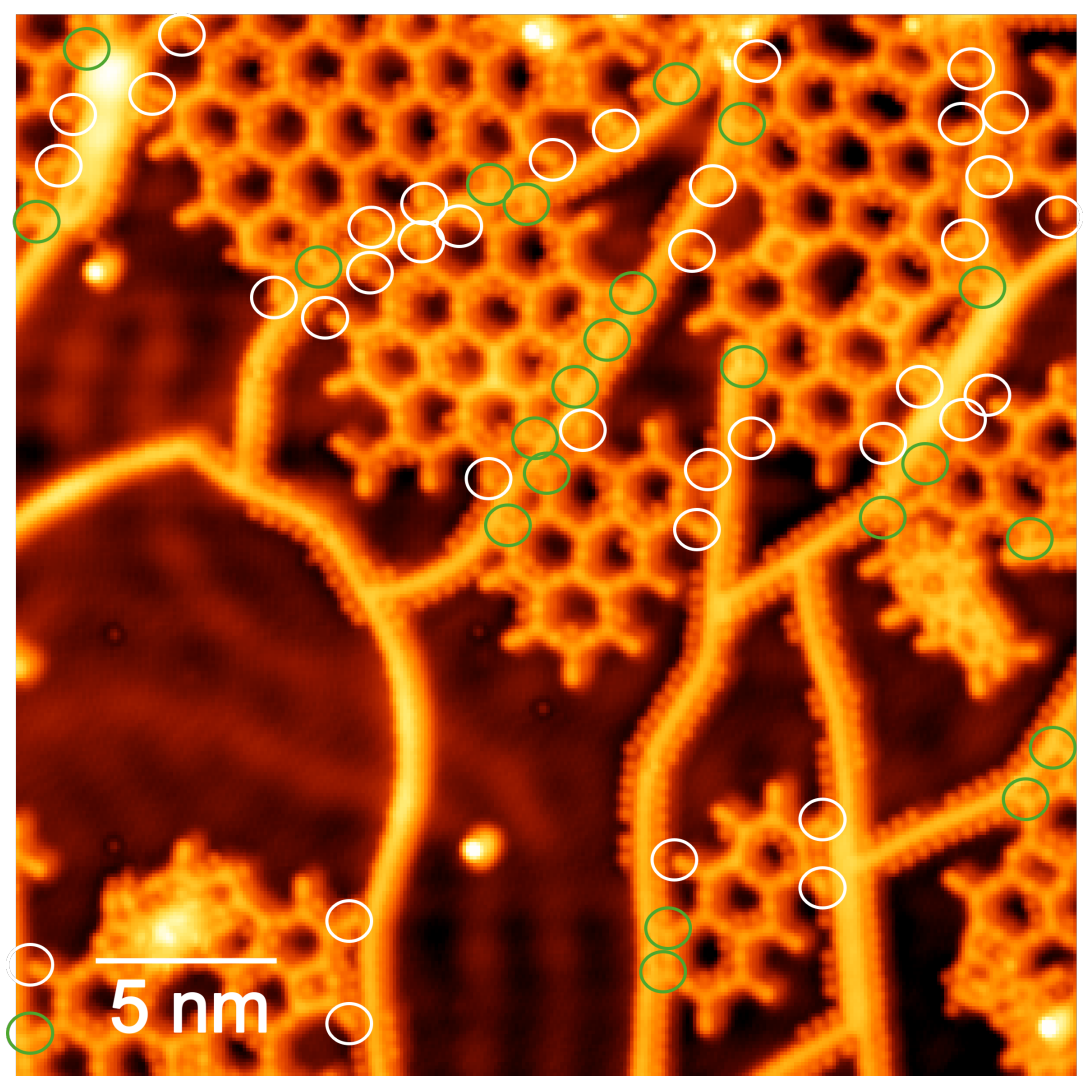

FIGURE S8 | High resolution LT-STM image of a region of the hGDY-aGNR heterostructure on Au(111) upon annealing at 530 K. Green circles represent the formed junctions. White circles represent the junctions which could have formed but have not formed. STM setpoint: −100 mV / 10 pA.

Sample 1 (standard tBEB coverage)

	Total possible linking sites	Number of linkages	Efficiency
Image #1	60	23	38.33%
Image #2	19	9	47.37%
Image #3	52	22	42.31%
	**TOT: 131**	**TOT: 54**	41.22%

The green circles (23) represent the junctions which have successfully formed, while the white circles (37) represent the sites where the junction could have possibly formed but has not formed, resulting in an efficiency of 38.33% for the linking process. The same process has been repeated for the other two LT-STM images, showing a linking efficiency of 47.37% (9 junctions formed over 19) and 42.31% (22 junctions formed over 52). Overall, 131 junctions have been considered, of which 54 have successfully formed, resulting in an efficiency of $\mathbf{41.22 \pm 4.30}$%, where the error bar is obtained as the standard error σ of Equation S1.
The mean linking efficiency obtained from the three individual LT-STM images is 42.67$\pm$4.5%, reflecting the reproducibility of the observation across different images. The same procedure has been applied to Samples 2 and 3.

Sample 2 (after hydrogen dosage)

	Total possible linking sites	Number of linkages	Efficiency
Image #1	30	27	90%
Image #2	48	31	64.58%
Image #3	66	40	60.61%
Image #4	27	18	66.67%
Image #5	34	20	58.82%
	**TOT: 205**	**TOT: 136**	66.34%

The efficiency is $\mathbf{66.34 \pm 3.30}$%. The efficiencies obtained from the five individual LT-STM images range from 58.82% to 90.00%, yielding a mean value of 68.13$\pm$12.6%. The relatively large spread reflects a more pronounced image-to-image variability of the linking efficiency compared to the other sample types. In particular, one image exhibits a significantly higher efficiency (90%) than the others, which contributes substantially to the observed standard deviation.

Sample 3 (increased tBEB coverage)

	Total possible linking sites	Number of linkages	Efficiency
Image #1	69	19	27.54%
Image #2	46	9	19.56%
Image #3	77	22	28.57%
Image #4	42	10	23.81%
Image #5	19	4	21.05%
Image #6	60	8	13.33%
	**TOT: 313**	**TOT: 72**	23%

The efficiency is $\mathbf{23 \pm 2}$%. The mean linking efficiency obtained from the six individual LT-STM images is 22.31$\pm$5.6%, reflecting the reproducibility of the observation across different images.

S8 | Atomic Hydrogen Dosage

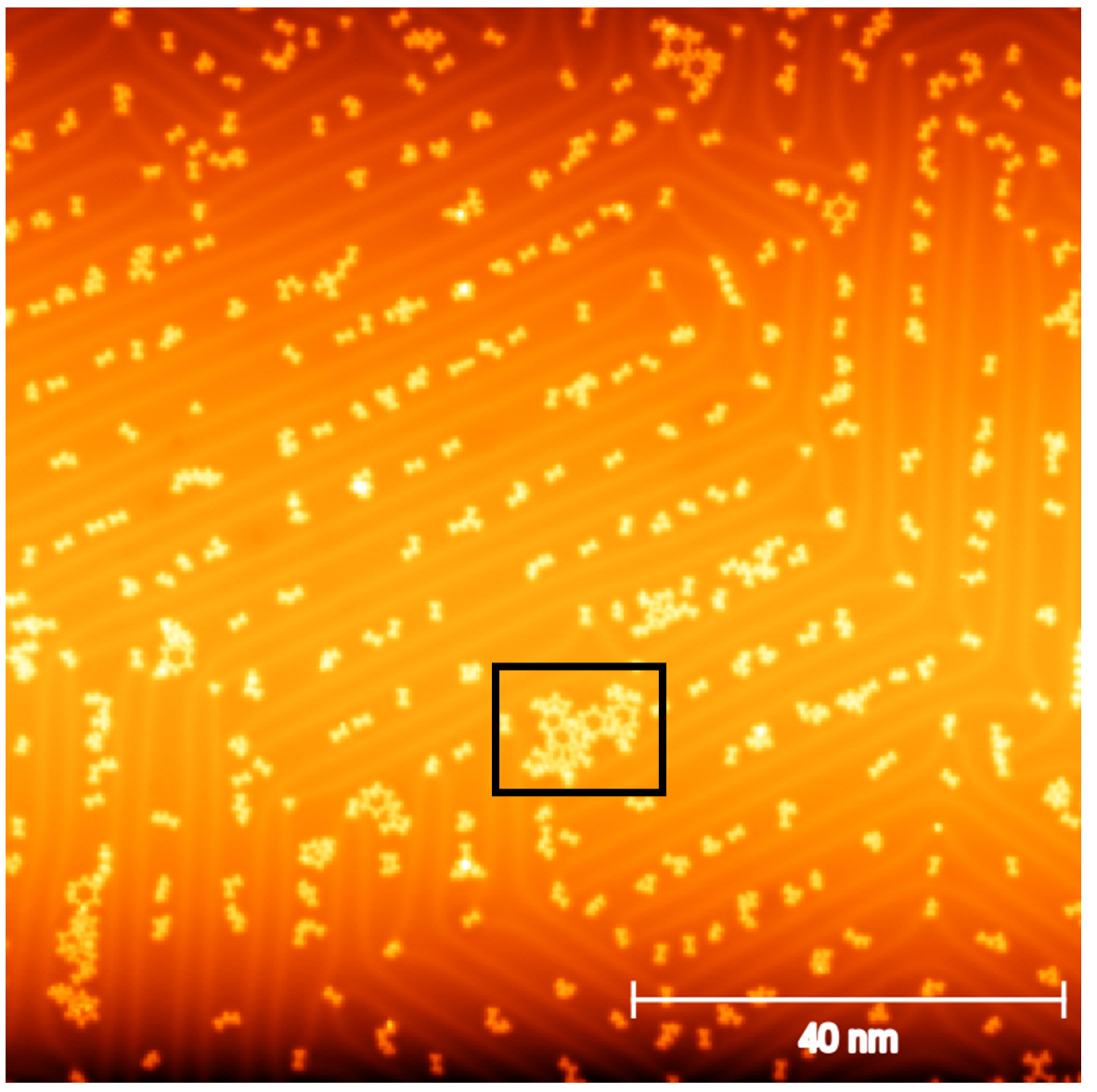

FIGURE S9 | Large-scale LT-STM image of tBEB molecules on Au(111) after atomic hydrogen dosage with a pressure of 1×10^{-7} mbar. Mainly tBEB dimers and just a small portion of extended network can be seen (black square). STM setpoint: 500 mV / 50 pA.

Even upon mild hydrogen dosage pressure at 2×10^{-8} mbar, the final system does not maintain the same degree of order as the pristine one, and some degradation effect can be observed, as shown in Figure S10, which compares the system before and after the atomic hydrogen dosage. Such irregularity is related to the reduction of Au-coordinated regions in the hGDY, which leads to uncontrolled covalent polymeric structures.

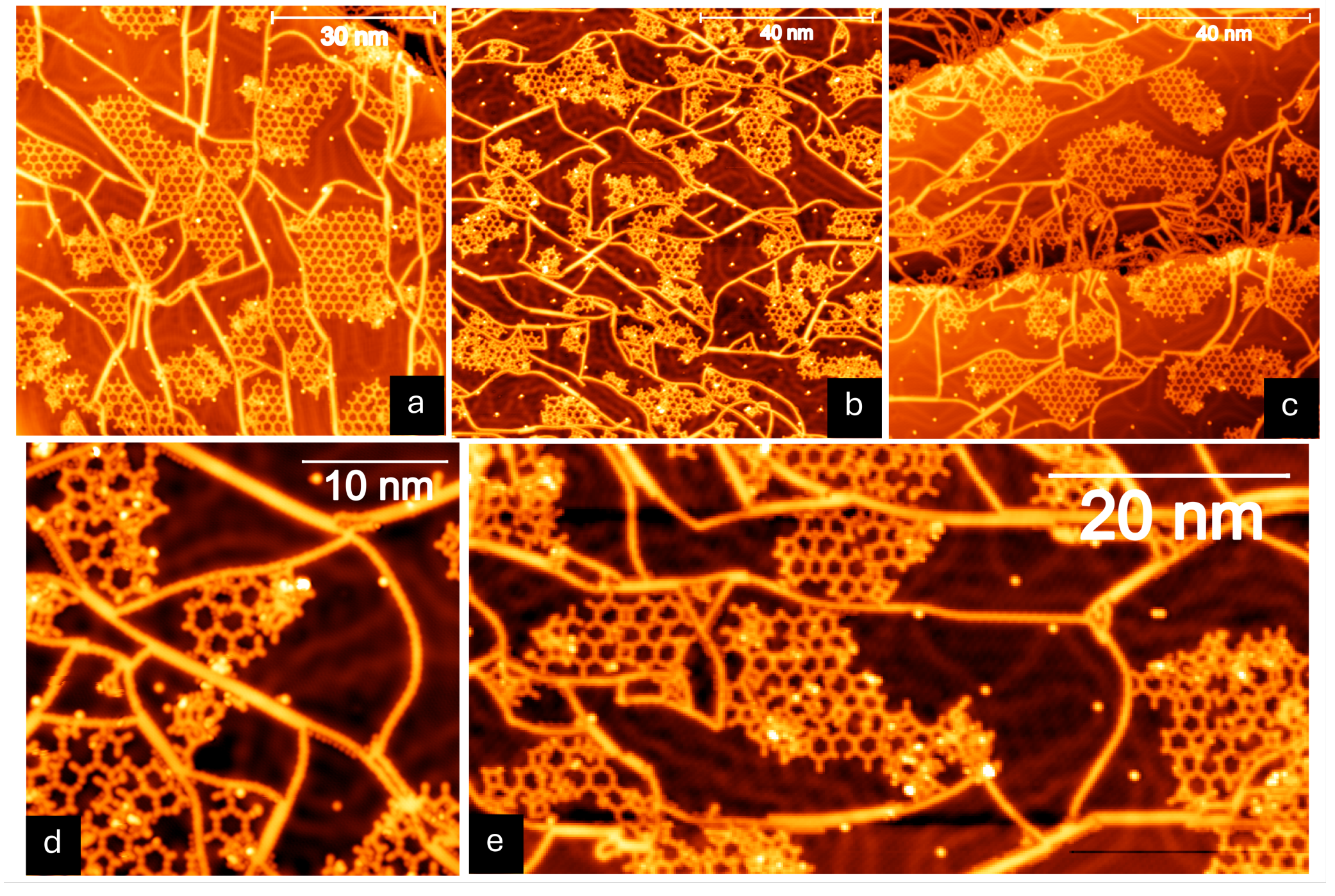

FIGURE S10 | Large-scale LT-STM images of the hGDY-aGNR heterostructure after the annealing at 530 K (a) and after the consequent atomic H dosage at RT, with a pressure of 2×10^{-8} mbar, and the following annealing at the same temperature, i.e. 530 K (b,c) (see Methods). High-resolution LT-STM images of disordered hGDY domains after H dosage (d,e). STM setpoints: (a) 300 mV / 50 pA, (b) −81 mV / 80 pA, (c) 500 mV / 50 pA, (d) 100 mV / 50 pA, (e) 100 mV / 50 pA.

S9 | PDOS on Selected Atoms in the Heterostructure

In Figure S11a and b, we report the PDOS on C atoms located at the linking site (C_1, C_2, C_3, with labels as reported in Figure 3f of the main text) and in the central region of hGDY and aGNR. From the comparison of these data it is evident that the features at −1.8 eV, 0.1 eV, and between 0.2 and 2.5 eV, in the green line of Figure S11a are localized states on C_3 atom that decay in the inner of the hGDY, as can be understood by their absence in the blue/cyan lines. The same conclusion holds for the state at the Fermi level in Figure S11b which is localized at the interface and decays in the inner of the aGNR (green line). These localized states do not contribute to electronic transport.

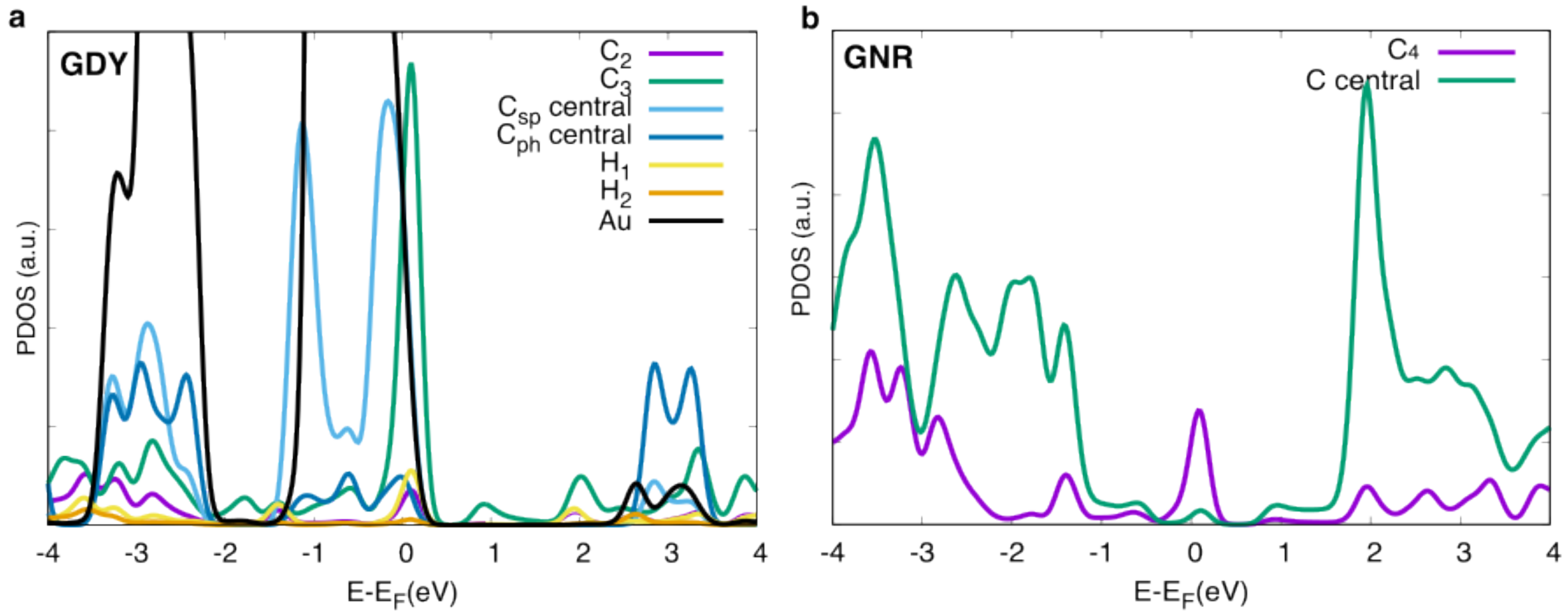

FIGURE S11 | PDOS on C atoms in different position of the hGDY (a) and aGNR in the freestanding "2H" heterostructure. In (a), the PDOS on H atoms at the linking site and Au adatoms in the hGDY are also reported.

S10 | Density of States of the Pristine hGDY and aGNR

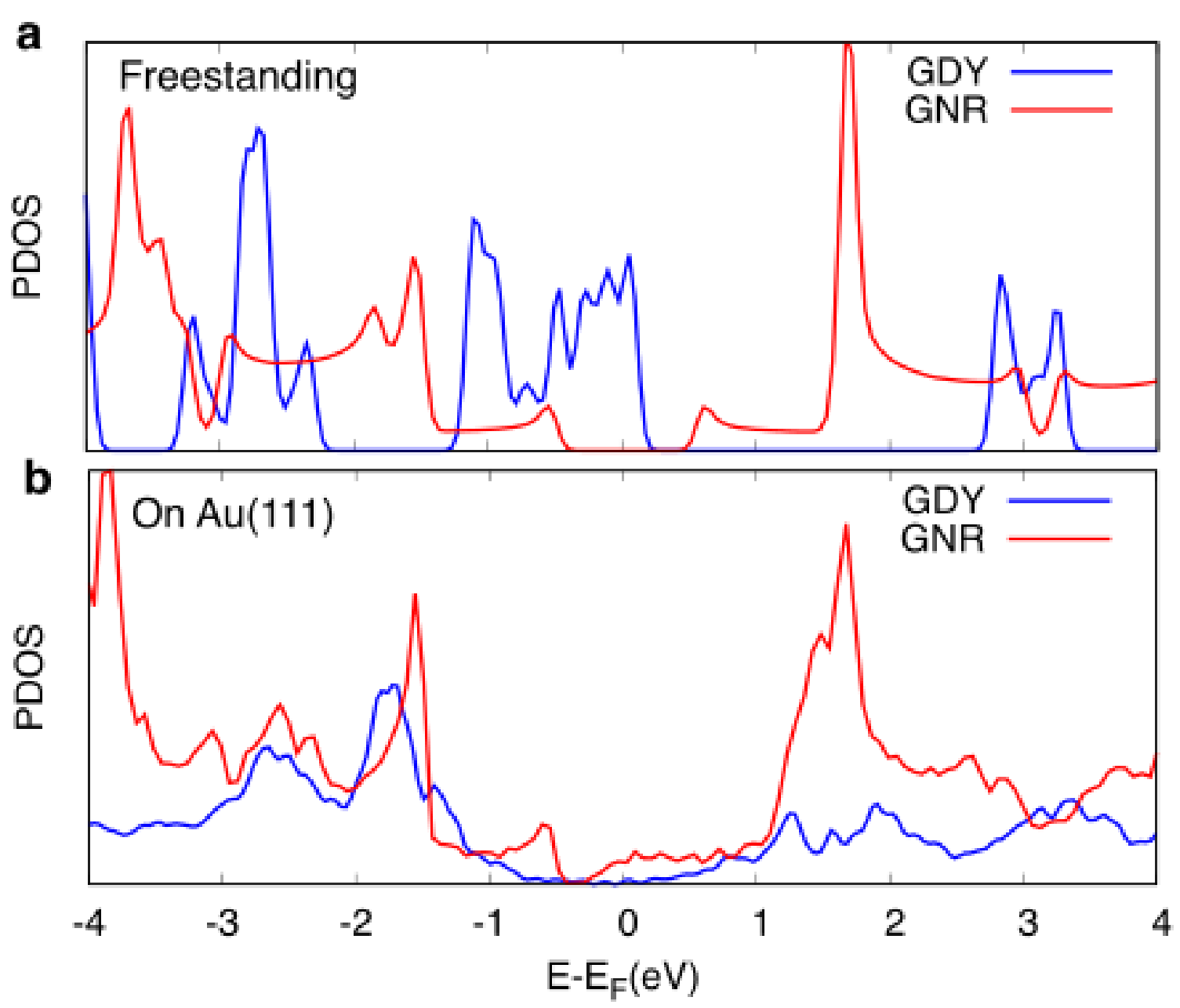

FIGURE S12 | PDOS of the freestanding (a) and Au(111) supported (b) hGDY and aGNR.

S11 | Additional Eigenchannels below the Fermi Level at −0.2 eV

In Figure S13, three additional eigenchannels with transmission probability nearly equal to 1, calculated near the Fermi level ($E = -0.2$ eV), are reported (the first eigenchannel is displayed in the main text). They are all localized on the hGDY.

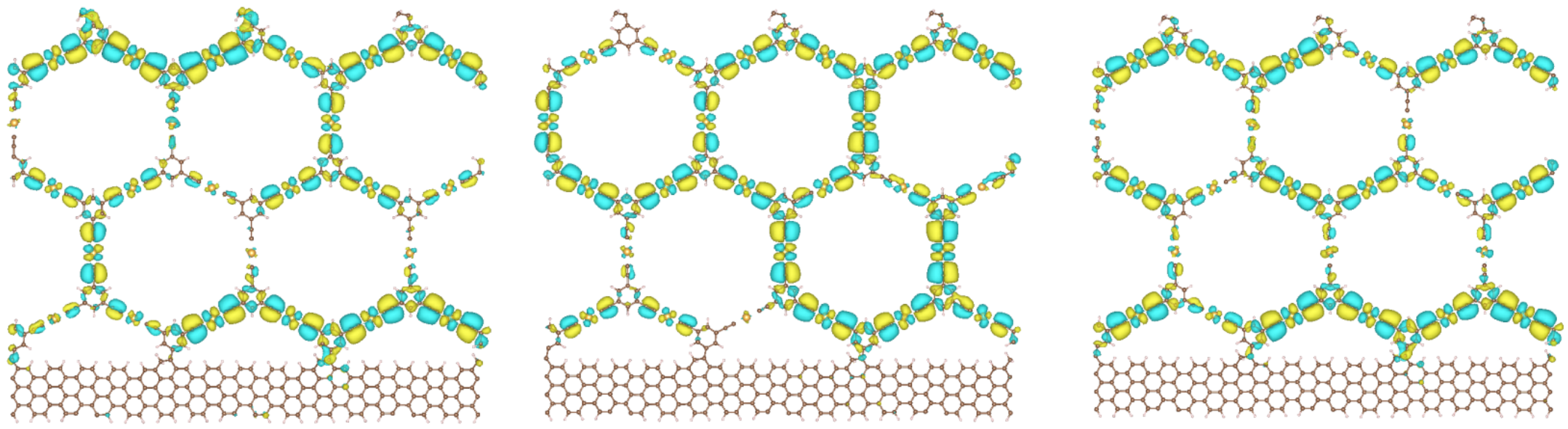

FIGURE S13 | Eigenchannels t_2, t_3 and t_4 for $E = -0.2$ eV.

S12 | PDOS and transmission of the "1H" freestanding configuration

In the freestanding case, the "1H" configuration is slightly less stable than "2H", unlike the supported case in which 1H is stabilized. For the sake of completeness, in Figure S14 the PDOS and transmission of the "1H" freestanding configuration are reported, for which the same conclusions reported in the main manuscript hold. In particular the separation of the electronic states of the two subsystems, with the presence of a gap just above the Fermi level, leads to eigenchannels that, depending on the energy, show different spatial distribution of the current in the heterojunction.

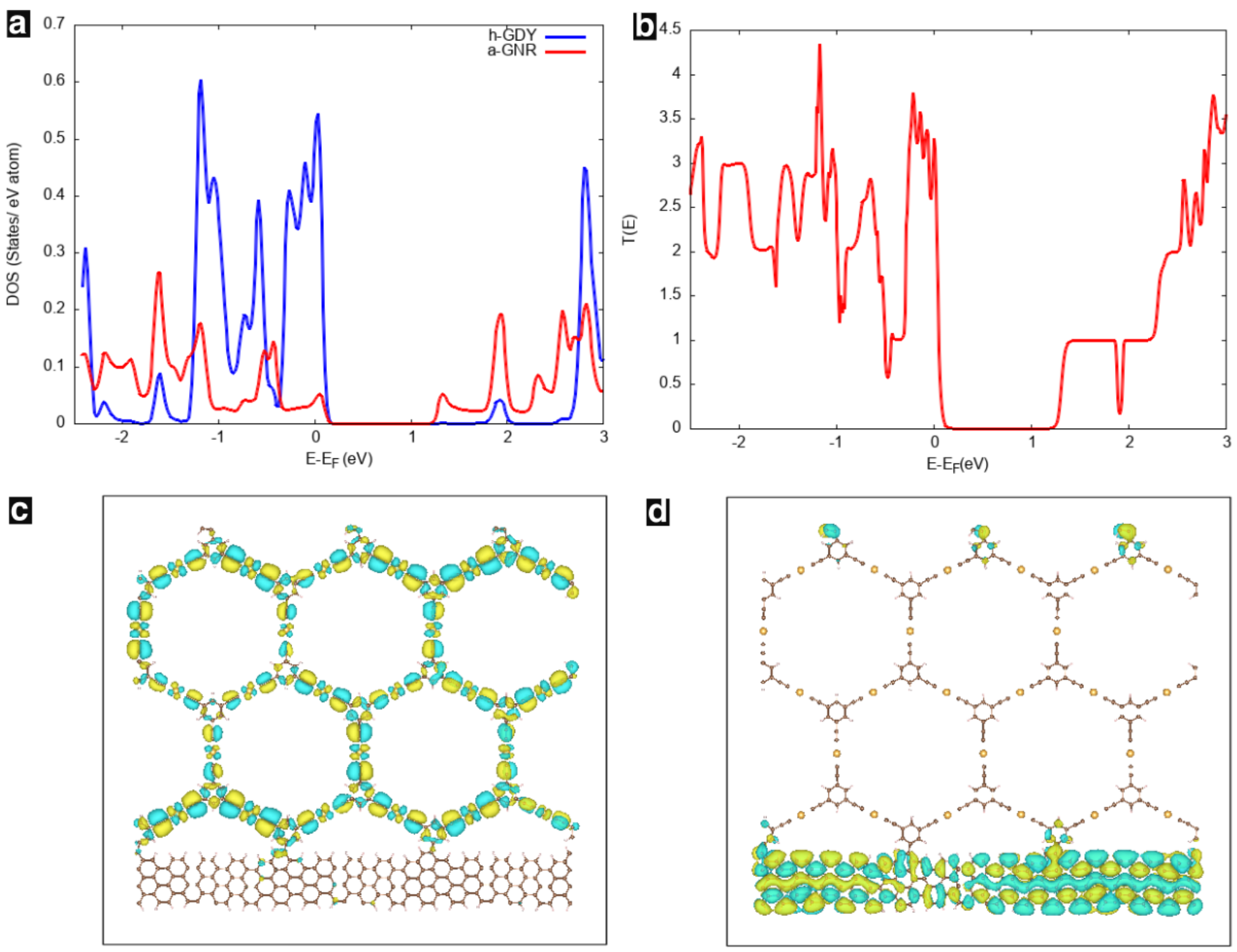

FIGURE S14 | (a) PDOS on aGNR (red) and hGDY (blue) carbon atoms. (b) Transmission of the freestanding "1H" heterojunction. (c) Eigenchannel at $E = -0.2$ eV. d) Eigenchannel at $E = 1$ eV.

S13 | hGDY Nucleation from aGNRs

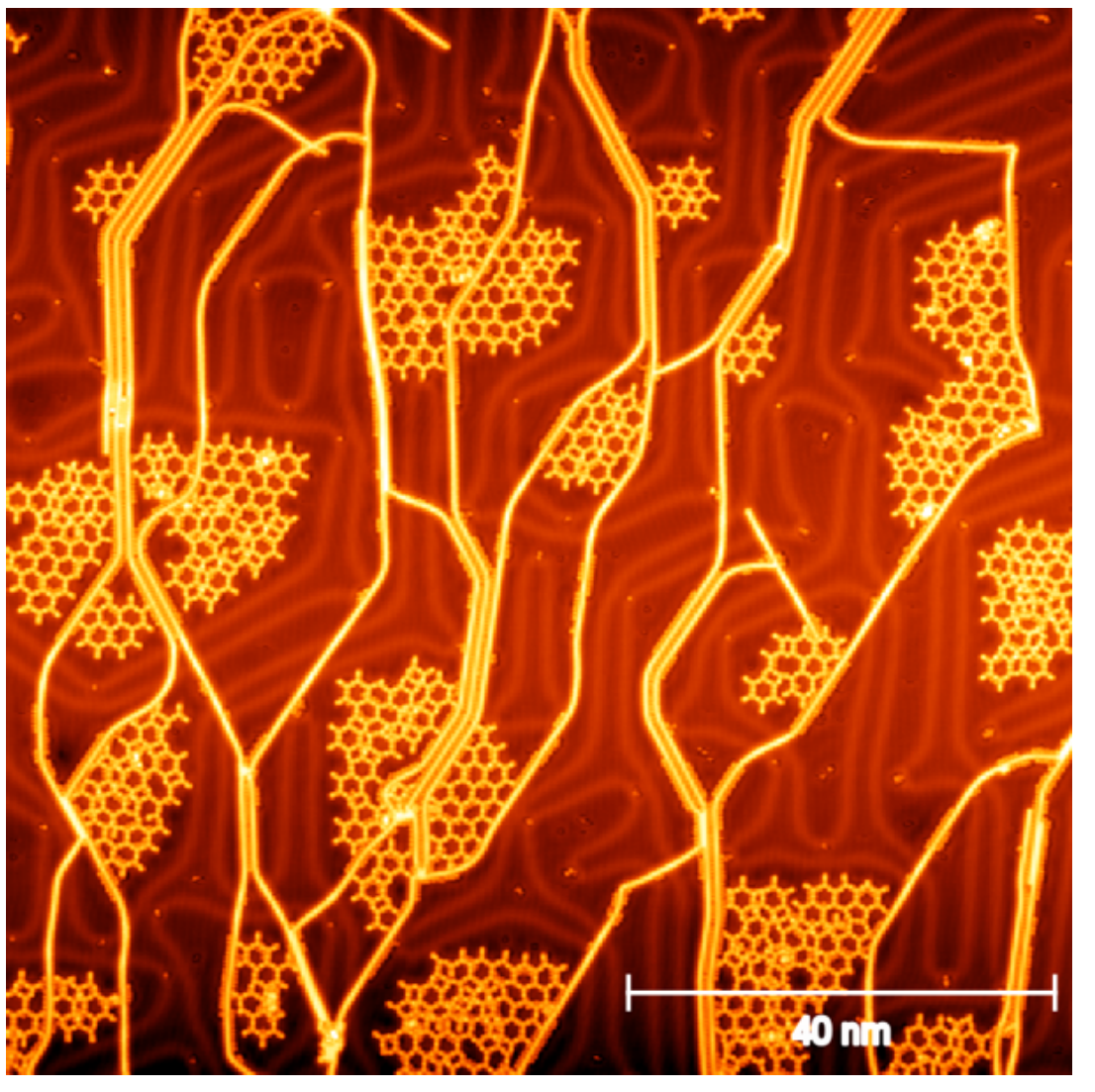

FIGURE S15 | Large-scale LT-STM image of the low coverage as-deposited hGDY-aGNR heterostructure. STM setpoint: 500 mV / 50 pA.
